# Clinicopathologic Features and Survival Outcomes of Signet Ring Cell Carcinoma of the Appendix: An Analysis of the Surveillance, Epidemiology, and End Results Database

**DOI:** 10.7759/cureus.8549

**Published:** 2020-06-10

**Authors:** Kamelah Abushalha, Wa'el Tuqan, Sara A Albagoush, Sawsan Abulaimoun, Peter T Silberstein

**Affiliations:** 1 Internal Medicine, Médecins Sans Frontières/Doctors Without Borders, Amman, JOR; 2 Department of Gastroenterology, Ochsner Health System, New Orleans, USA; 3 Internal Medicine, CHI Creighton University Medical Center, Omaha, USA; 4 Department of Medicine, Creighton University School of Medicine, Omaha, USA; 5 Oncology, Creighton University School of Medicine, Omaha, USA

**Keywords:** signet cell histology, appendiceal tumor, survivorship, seer

## Abstract

Background and objective

Signet ring cell carcinoma of the appendix (SRCCA) is an exceedingly rare tumor, and very limited data are available regarding its characteristics and survival probabilities. Our objective in this study was to utilize the Surveillance, Epidemiology, and End Results (SEER) database to explore the patient and tumor characteristics and to characterize the three- and five-year cancer-specific survival (CSS) probabilities of SRCCA.

Methods

Patients with SRCCA diagnosed between 2000 and 2015 were analyzed using the SEER database. The three- and five-year CSS probabilities were estimated by the Kaplan-Meier method, and the groups were compared using log-rank comparisons and multivariable Cox hazard regression analysis.

Results

A total of 527 patients were identified. The median age of the participants at diagnosis was 56 years, with a majority of them being female and white. Histologically, 60% of the tumors were high grade, and 61.3 % of the tumors were found to be metastatic on presentation. Three- and five-year CSS probabilities were 39% and 18.4%, respectively, and median survival was 26 months. Best survival outcomes were noted in males (five-year CSS: 25.4%, p=0.027), unmarried patients (five-year CSS: 19.1%, p=0.042), tumors <2 cm in size (five-year CSS: 50.5%, p<0.001), and low-grade tumors (five-year CSS: 44.8%, p<0.001). Subtotal colectomy yielded better three- and five-year CSS probabilities compared to no surgery and partial colectomy (48.5% and 26.5%, respectively, p<0.001). On the multivariate analysis, it was found that age and stages T4, N1, and M1 were associated with an increased risk of mortality, while surgery, regardless of the extent, was a protective factor.

Conclusion

SRCCA is a rare tumor with a high prevalence among old-aged white females. This tumor is usually diagnosed in an advanced stage and has a dismal prognosis. Surgical intervention, regardless of the extent, showed better survival probabilities compared to no surgery.

## Introduction

Cancers of the appendix are very unusual, accounting for only about 0.4% of all gastrointestinal (GI) malignancies [1]. Furthermore, signet ring cell carcinoma of the appendix (SRCCA) is an exceedingly rare entity. SRCC has been defined by the World Health Organization classification as adenocarcinoma in which the predominant component (more than 50%) is composed of isolated malignant cells containing intracytoplasmic mucin [2]. Case reports and case series suggest that SRCCA is highly aggressive and has a dismal prognosis [3-5]; however, limited data are available on the discrete characteristics and survival probabilities of this tumor. We aimed to utilize the Surveillance, Epidemiology, and End Results (SEER) database to explore patient and tumor characteristics and to characterize the three- and five-year cancer-specific survival (CSS) probabilities of SRCCA. We also engaged in a review of the current management approaches related to SRCCA. Since SEER is a de-identified, publicly available database, the National Cancer Institute does not mandate institutional review board approval for SEER studies.

## Materials and methods

SRCCA data extracted from the SEER database [Incidence - SEER 18 Regs Research Data + Hurricane Katrina Impacted Louisiana Cases, Nov 2017 Sub (2000-2015)] were employed to perform this population-based study from January 2000 to December 2015. Primary tumor site was chosen as “Appendix”, year of diagnosis selected was “2000 to 2015”, and site records of International Classification of Diseases (ICD) codes, third version (ICD-0-3) was used to identify SRCC “8490/3”. Tumors that were not microscopically confirmed were excluded. Patients with incomplete survival data or patients with more than one primary tumor were also excluded. No one in our cohort was diagnosed by autopsy and death certificate.

The following primary data were drawn from the database for analysis: year of diagnosis, age at diagnosis, sex, marital status, race, tumor size, tumor grade, the American Joint Committee on Cancer (AJCC) staging system, distant metastasis at the time of diagnosis, surgery of the primary site, cause of death, and survival time. Well-differentiated and moderately-differentiated histologic features were defined as low-grade, while poorly differentiated and undifferentiated histologic types were classified as high-grade. The AJCC Staging Manual third edition was used for tumors diagnosed within the period (2000 through 2004) and the sixth edition was used for tumors diagnosed since 2004. Primary site surgery types were as follows: 1 - no surgery performed (patients who underwent local tumor destruction or excision were included within this category); 2 - partial colectomy (but less than hemicolectomy); 3 - subtotal colectomy; and 4 - total colectomy. No specific code for appendectomy exists in the SEER, but the partial colectomy code reflects and is defined as the performance of an appendectomy.

SEERStat version 8.3.5 was used to search for relevant cases. SPSS Statistics version 23 (IBM, Armonk, NY) and SAS version 9.4 (SAS Institute, Cary, NC) were used for statistical analysis. Descriptive statistics were computed for all variables. Median survival and three- and five-year CSS probabilities for all variables were calculated in months from the time from diagnosis until either death or last known contact. To calculate three- and five-year survival probabilities, survival tables, and Kaplan-Meier curves were utilized. Multivariable Cox regression model was used to calculate the mortality-associated risk factors in those with SRCCA after adjusting for a series of indexes. Deviations between groups were considered statistically significant at a p-value of <0.05.

## Results

We included 527 patients with SRCCA (337 women and 190 men). Table 1 illustrates the demographic and tumor-related characteristics of the participants. Women accounted for 63.9% of patients. The mean (SD) age at diagnosis was 56 (12.69) years (range: 27-94 years). Approximately 64% of the included patients were married, and 83.8% were white. Overall, 60% of the tumors were histologically confirmed to be poorly differentiated or undifferentiated tumors of high grade. Tumors invading adjacent organs or those that had grown through the visceral peritoneum accounted for 53.7% of the sample. Over 44% had SRCCA metastasizing to regional lymph nodes, and tumors metastasizing to distant locations made up 61.3% of the sample. The mean and median tumor size at the time of diagnosis was 45.4 and 40 mm, respectively; 87% of the patients had tumors equal to or larger than 20 mm.

A total of 456 patients (86.5%) underwent surgery, ranging from partial colectomy to total colectomy. Surgery stratified by stage is shown in Table 2. The most common surgery performed was subtotal colectomy (208 out of 456 patients, 45.6%), followed by partial colectomy (153 out of 456 patients, 33.6%), and total colectomy (81 out 456 patients, 17.8%).

**Table 1:** Cohort characteristics NOS: not otherwise specified; SD: standard deviation; n: number of patients

Characteristic	Total		Female		Male	
Patients, n	527		337	63.90%	190	36.10%
Age, years						
Median	56		56		56	
Mean (SD)	57.22 (12.69)		57.05 (12.85)		57.52 (12.43)	
Marital status, n						
Married	336	63.8%	202	59.9%	134	70.5%
Unmarried	172	32.6%	120	35.6%	52	27.4%
Unknown	19	3.6%	15	4.5%	4	2.1%
Race, n						
White	439	83.3%	284	84.3%	155	81.6%
Black and other	87	16.5%	52	15.4%	35	18.4%
Unknown	1	0.2%	1	0.3%		
Tumor grade, n						
High grade	316	60%	204	60.5%	112	58.9%
Low grade	41	7.8%	26	7.7%	15	7.9%
Unknown	170	32.3%	107	31.8%	63	33.2%
Tumor size, mm						
Median	40		40		42	
Mean (SD)	45.4 (27.77)		44.38 (29.22)		46.95 (25.45)	
Primary tumor, n						
T1	13	2.5%	8	2.4%	5	2.6%
T2	6	1.1%	4	1.2%	2	1.1%
T3	140	26.6%	80	23.7%	60	31.6%
T4	283	83.9%	184	54.6%	99	52.1%
Tx	85	16.1%	61	18.1%	24	12.6%
Regional lymph node, n						
N0	209	39.7%	130	38.6%	79	41.6%
N1	235	44.6%	146	43.3%	89	46.8%
Nx	83	15.7%	61	18.1%	22	11.6%
Metastasis, n						
M0	199	37.8%	98	29.1%	101	53.2%
M1	323	61.3%	235	69.7%	88	46.3%
Mx	5	0.9%	4	0.2%	1	0.5%
Surgery, n						
No surgery	71	13.5%	49	14.5%	22	11.6%
Partial colectomy	153	29%	95	28.2%	58	30.5%
Subtotal colectomy	208	39.5%	113	33.5%	95	50%
Total colectomy	81	15%	70	20.8%	11	5.8%
Surgery NOS	14	2.7%	10	3%	4	2.1%

**Table 2:** Surgery type stratified by stage NOS: not otherwise specified; n: number of patients

Stage	No surgery	Partial colectomy	Subtotal colectomy	Total colectomy	Surgery NOS
Stage I, n	2	3	6	0	2
Stage II, n	3	38	40	7	3
Stage III, n	0	16	63	9	0
Stage IV, n	61	90	99	65	8
Unspecified, n	5	6	0	0	1
Total, n (%)	71 (13.47%)	153 (29.03%)	208 (39.48)	81 (15.37%)	14 (2.65%)

Table 3 demonstrates the results of multivariate Cox proportional hazards analyses for the mortality-associated risk factors in patients with SRCCA. Age [hazard ratio (HR) = 1.02, 95% CI: (1.00, 1.03)], T4 [HR = 1.96, 95% CI: (1.29, 2.97)], N1 [HR = 1.90, 95% CI: (1.30, 2.77)], and M1 [HR = 2.62, 95% CI: (1.76, 3.91)] were associated with an increased risk of mortality, while surgery, regardless to the extent, was a protective factor for survival: partial colectomy [HR = 0.45, 95% CI: (0.25, 0.82)], subtotal colectomy [HR = 0.51, 95% CI: (0.28, 0.93)] and total colectomy [HR = 0.47, 95% CI: (0.24, 0.94)].

**Table 3:** Multivariate analysis results AJCC: American Joint Committee on Cancer; CI: confidence interval

	Hazard ratio	95% CI lower limit	95% CI upper limit	P-value
Age, years	1.02	1	1.03	0.051
Females vs. males	1.33	0.96	1.86	0.088
AJCC T category: T4 vs. T1	1.96	1.29	2.97	0.002
AJCC N category: N1 vs. N0	1.9	1.3	2.77	<0.001
AJCC M category: M1 vs. M0	2.62	1.76	3.91	<0.001
Surgical treatment				
Partial colectomy vs. no surgery	0.45	0.25	0.82	0.008
Subtotal colectomy vs. no surgery	0.51	0.28	0.93	0.028
Total colectomy vs. no surgery	0.47	0.24	0.94	0.031

**Figure 1:**
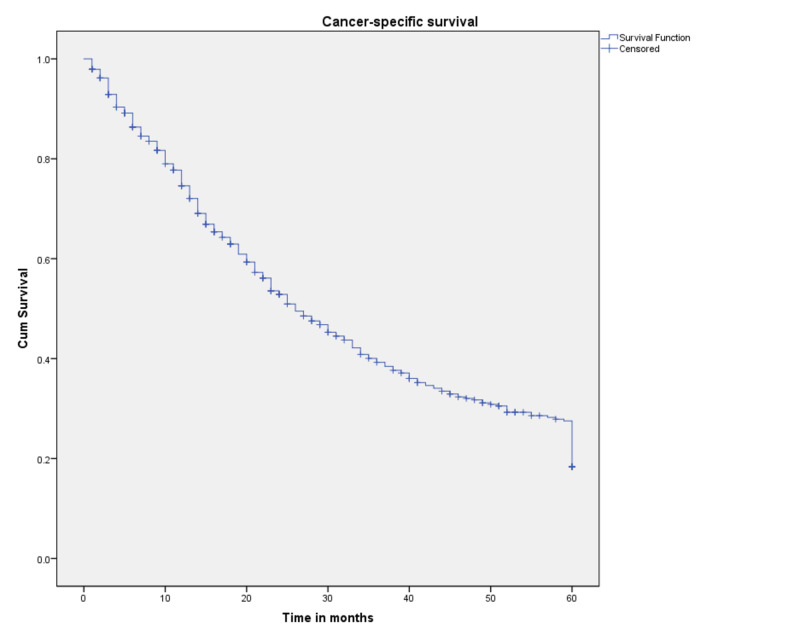
Cancer-specific survival for patients with SRCCA (n=527) SRCCA: signet ring cell carcinoma of the appendix; cum: cumulative

**Figure 2:**
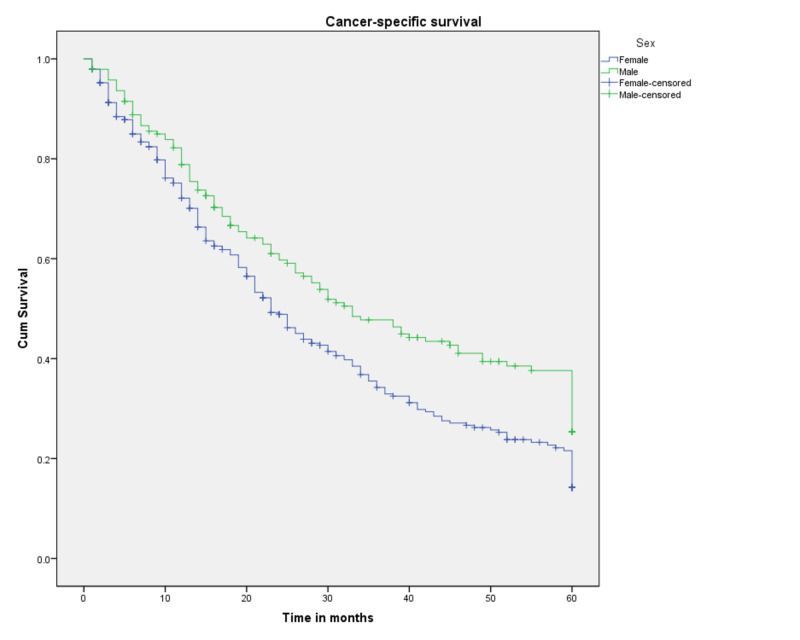
Cancer-specific survival for patients with SRCCA by sex (n=527, p<0.002) SRCCA: signet ring cell carcinoma of the appendix; cum: cumulative

Three and five-year CSS probabilities for the 527 patients with SRCCA were 39% and 18.4%, respectively, and median survival was 26 months. A Kaplan-Meier curve for the cohort as a whole is shown in Figure 1. A Kaplan-Meier curve by sex is shown in Figure 2. Females showed three- and five-year CSS probabilities of 34.2% and 14.2%, respectively, while males exhibited three- and five-year CSS probabilities of 47.7% and 25.4%, respectively. Both race and marital status were not found to be associated with a statistically significant difference in survival.

**Figure 3:**
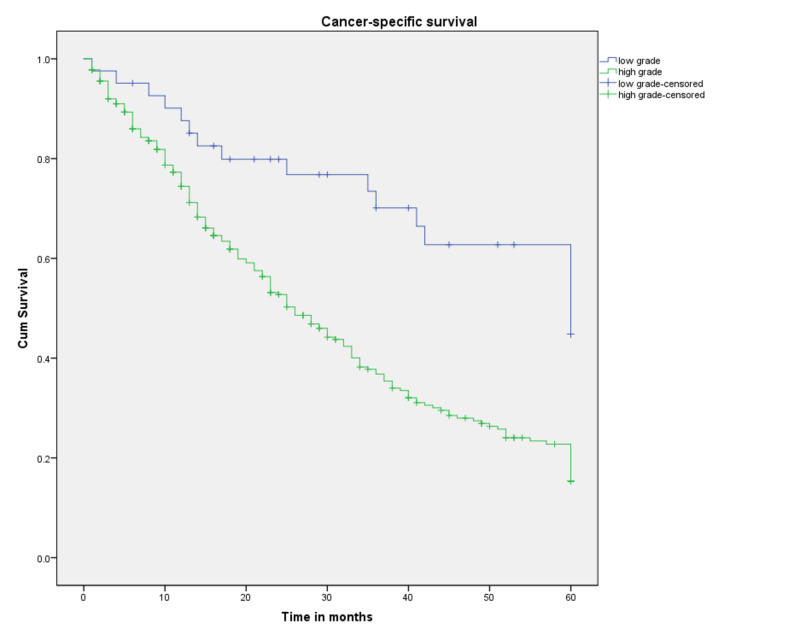
Cancer-specific survival for patients with SRCCA by tumor grade (n=358, p<0.001) SRCCA: signet ring cell carcinoma of the appendix; cum: cumulative

Survival probabilities by tumor grade are shown in Figure 3. Low-grade tumors (well-differentiated and moderately differentiated) are shown to have significantly better three- and five-year CSS probabilities compared to high-grade tumors (poorly differentiated and anaplastic); (70.1% and 44.8% vs. 36.8% and 15.4%, respectively). The median survival for low-grade tumors was 60 months, which was more than double the value for high-grade tumors (26 months, p<0.001).

**Figure 4:**
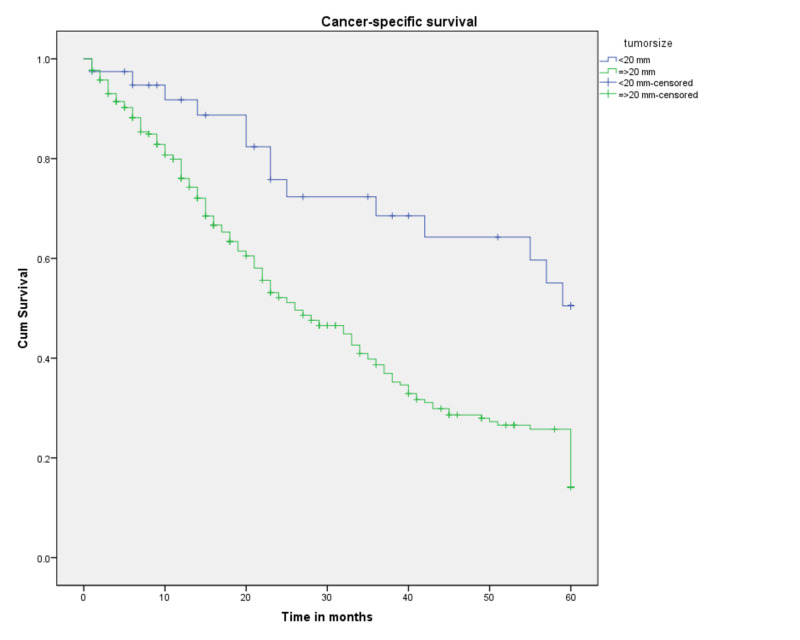
Cancer-specific survival for patients with SRCCA by tumor size (n=301, p<0.001) SRCCA: signet ring cell carcinoma of the appendix; cum: cumulative

Survival probabilities by tumor size are graphed as a Kaplan-Meier curve in Figure 4. Tumor size was found to significantly affect survival probabilities, with tumors less than 20 mm having better three- and five-year survival probabilities (68.5% and 50.5%, respectively) compared to tumors greater than or equal to 20 mm (38.7% and 14%, respectively) (p<0.001).

**Figure 5:**
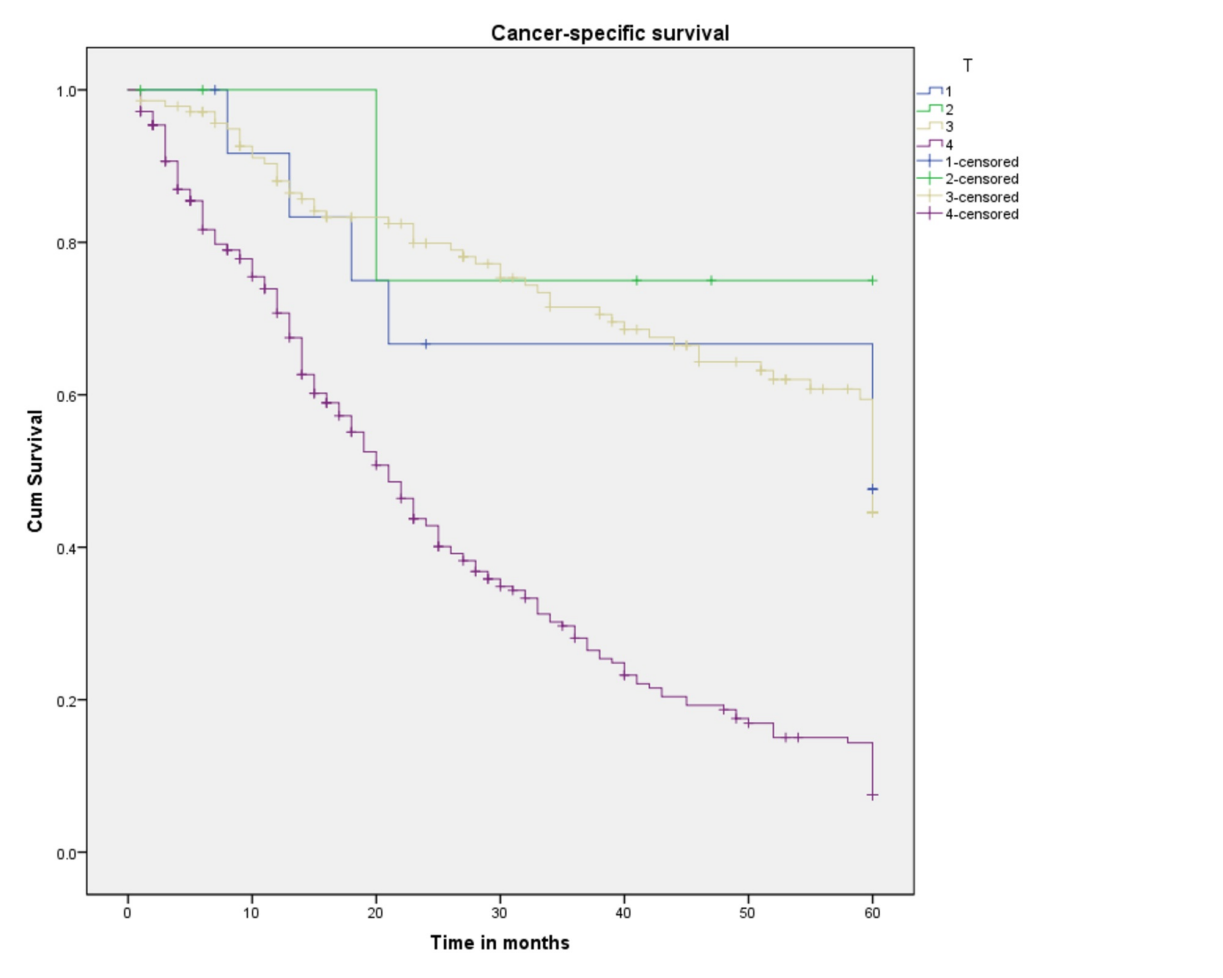
Cancer-specific survival for patients with SRCCA by AJCC TNM T category (n=442, p<0.001) SRCCA: signet ring cell carcinoma of the appendix; AJCC: American Joint Committee on Cancer; cum: cumulative

**Figure 6:**
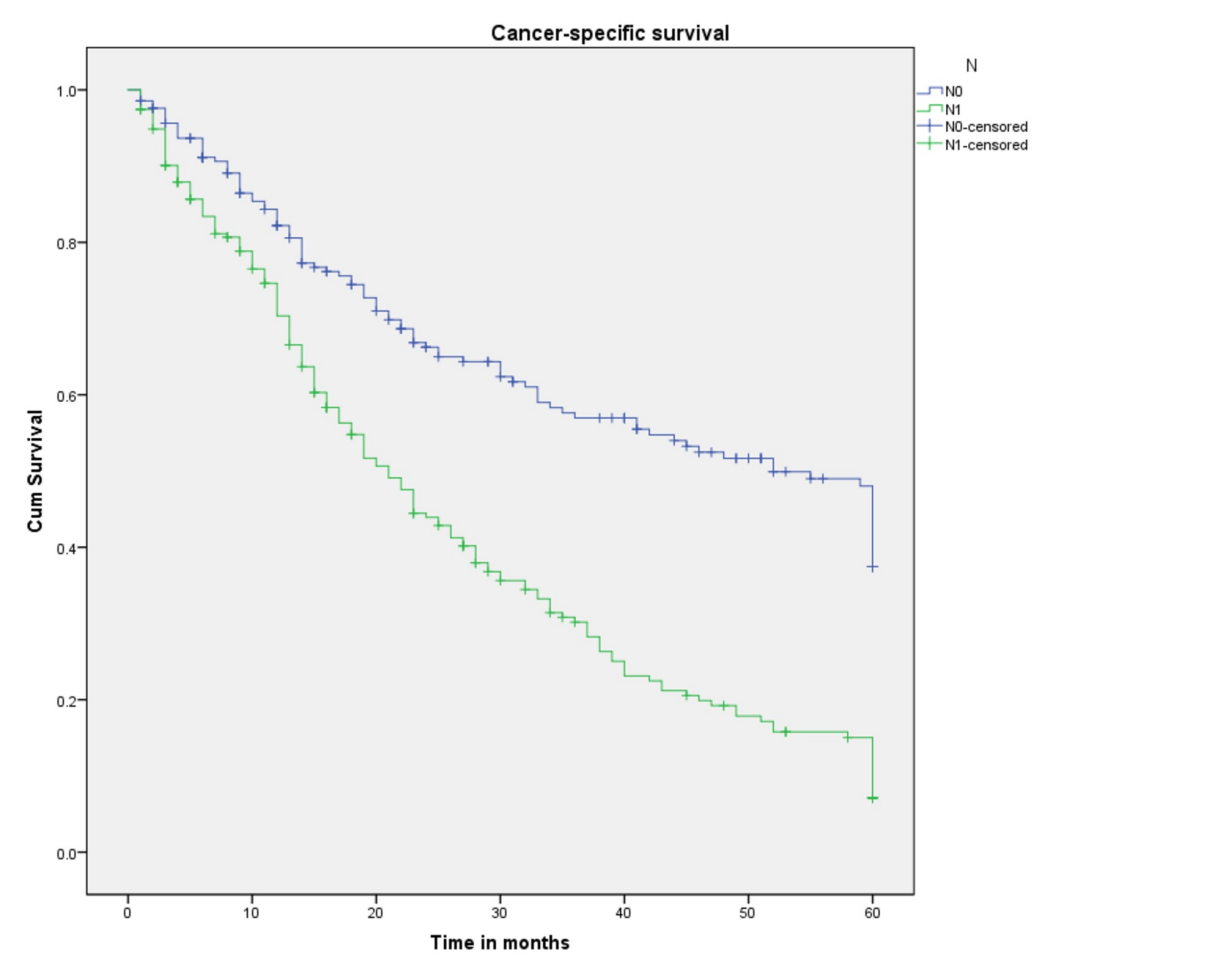
Cancer-specific survival for patients with SRCCA by AJCC TNM N category (n=444, p<0.001) SRCCA: signet ring cell carcinoma of the appendix; AJCC: American Joint Committee on Cancer; cum: cumulative

**Figure 7:**
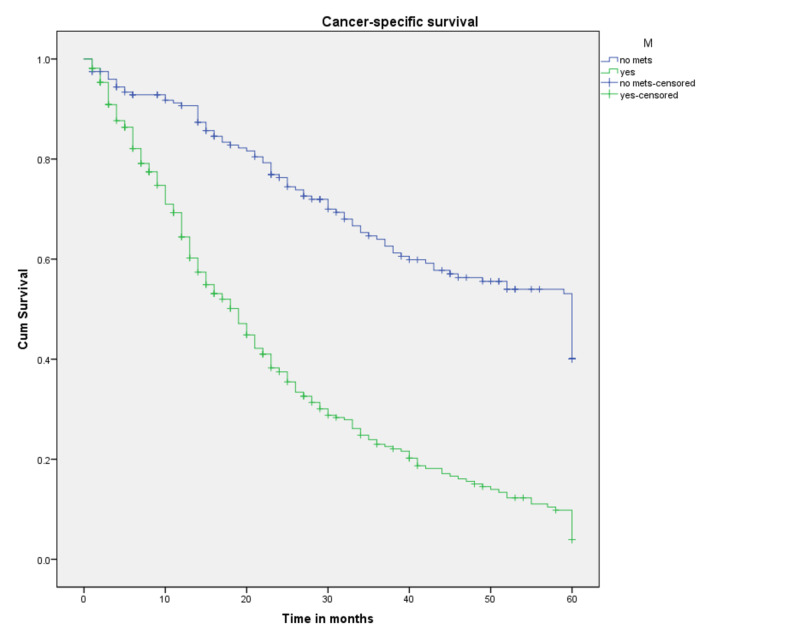
Cancer-specific survival for patients with SRCCA by AJCC TNM M category (n=522, p<0.001) SRCCA: signet ring cell carcinoma of the appendix; AJCC: American Joint Committee on Cancer; cum: cumulative

Survival probabilities by each component of the TNM classification are plotted as Kaplan-Meier curves in Figures 5-7. As expected, T4, N1, and M1 had the worst survival probabilities. Table 4 lays out the detailed results. It is worth noting that the sample size for T1 and T2 accounted for the inability to estimate the median survival and survival probabilities. While three- and five-year CSS probabilities for T3 were 70% and 44.5%, respectively, for T4 there was a significant drop, 28.1% and 7.5%, respectively (p<0.001). Regional lymph node involvement (N1) showed a median survival of 21 months (p<0.001), while that for a patient with distant metastasis (M1) was 19 months (p<0.001).

**Table 4:** Three- and five-year survival probabilities a: couldn’t be calculated *Survival at 35 months

Variable	Probability of three-year survival (%)	Probability of five-year survival (%)	Median survival (months)
Overall	39.3	18.4	26
Sex			
Female	34.2	14.2	23
Male	47.7	25.4	33
Race			
White	39	18	26
Black and other	34.5	19	27
Marital status			
Married	40.4	17.2	27
Unmarried	34.5	19.1	23
Grade			
Low grade	70.1	44.8	60
High grade	36.8	15.4	26
Tumor size			
<20 mm	68.5	50.5	a
≥20 mm	38.7	14	26
Primary tumor			
T1	A	a	a
T2	a	a	a
T3	70	44.5	60
T4	28.1	7.5	21
Regional lymph node			
N0	57	37.5	52
N1	30.2	7.1	21
Metastasis			
M0	64	40	60
M1	23	3.9	19
Surgery extension			
Partial colectomy	44.9*	20.9	33
Subtotal colectomy	48.5	26.5	35
Total colectomy	24.4	3.4	19
No surgery	12.5*	6.2	13

**Figure 8:**
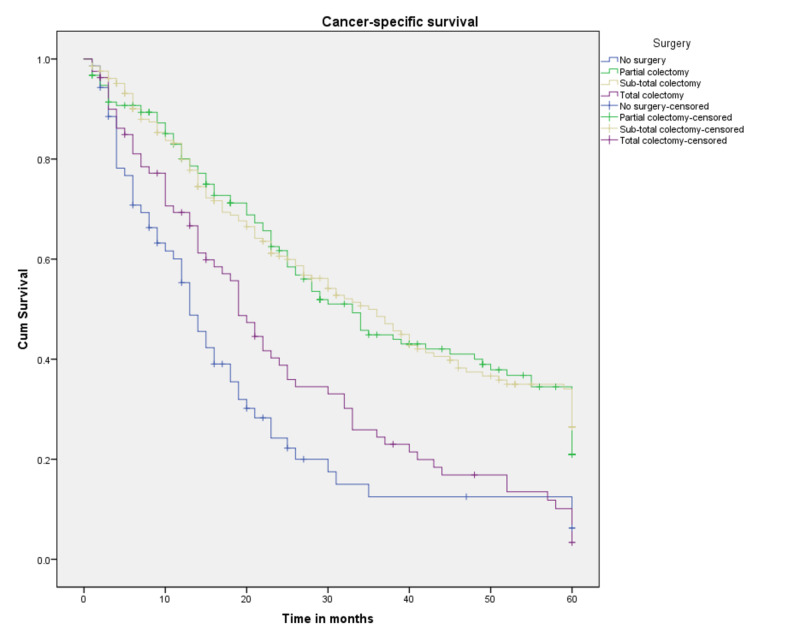
Cancer-specific survival for patients with SRCCA by surgical treatments (n=513, p<0.001) SRCCA: signet ring cell carcinoma of the appendix; cum: cumulative

Figure 8 represents Kaplan Meier curves for different surgical treatments; all surgical interventions showed better CSS and median survival time compared to no surgery. Of all the surgical methods, subtotal colectomy had the highest median survival (35 months) and the best three- and five-year CSS (48.5% and 26.5%, respectively; p<0.001), followed by partial colectomy with a median survival of 33 months and three- and five-year survival probabilities of 44.9% and 20.9%, respectively (p<0.001). Total colectomy resulted in the worst three- and five-year survival outcomes at 24.4% and 3.4%, respectively (p<0.001), and the worst median survival (19 months).

## Discussion

Case reports and case series constitute most of the published literature on primary SRCCA. To the best of our knowledge, only three previously published studies have utilized the SEER database to address the appendiceal malignancies. McCusker et al. (2002) analyzed the demographic characteristics of patients with cancer of the appendix reported to the SEER database between 1973 and 1998; the study included 70 cases of SRCCA [6]. The second study was done by Turaga et al. (2012) in which they compared the incidence and survival for the different histological subtypes of appendiceal cancers; the study covered the cohort of cases reported to the SEER between 1973 and 2007 and included 313 patients with SRCCA [7]. The third study by McGory at el. (2005) was specific to the management and prognosis of adenocarcinoma of the appendix, and cases reported to SEER between 2004 and 2013 were included; According to the study, the adenocarcinoma included three subtypes: mucinous, non-mucinous, and signet cell; no specific numbers and statistics were given pertaining to each subtype [8]. All of these studies emphasized the poor prognosis of SRCCA compared to other histologies. Due to the paucity of published research on this notorious cancer, we aimed to focus our study specifically on SRCCA to analyze the specific tumor and patient characteristics and to give survival statistics. Our cohort consisted of a total of 456 patients diagnosed between 2000 and 2015.

Turaga et al.'s study (2012) showed a female-sex predominance (60%), which is consistent with our results (64%) [7]. Although females have a worse CSS probability compared to males, such a difference was lost on the multivariate analysis. The majority of patients in our analysis were white, a finding consistent with all different histological subtypes of appendiceal malignancies [6-8]. Nevertheless, no statistically significant difference in survival was found based on race.

The effect of tumor size on survival for appendiceal malignancies has been examined. McGory at el. have stated that there is a statistically significant drop in five-year survival with increasing tumor size for noncarcinoid tumors in general (78% for tumors less than or equal to 1 cm vs. 43% for tumors more than 2 cm; p=0.04) and addressed the need for a more aggressive treatment approach (right hemicolectomy) rather than simple appendectomy [8]. In our study, the five-year CSS for SRCCA showed a significant difference based on size, but such a difference was lost in the multivariate analysis, which may be due to the fact that the size of tumors for a lot of cases was not reported and was subsequently excluded from the analysis.

In a study addressing the importance of histological subtype in the staging of appendiceal tumors, Turaga et al. reported a median survival of 24 months for patients with SRCCA [7]; our median survival was very close (26 months). Another study reported five-year overall survival rates of 18% and 7%, for all stages and for metastatic disease, respectively [8]. In our analysis, the five-year CSS for all stages was 18.4, and for metastatic disease, it was 4%. Metastasis at presentation is more common for SRCCA than other histological subtypes. In the aforementioned three SEER studies, the percentage of metastatic disease at presentation was as follows; 60%, 76%, and 56% [6-8]. In our analysis, the percentage of metastatic disease fell between the percentages in previous studies, accounting for 61% of the cases.

SRCCA is managed similarly to other subtypes of appendiceal adenocarcinomas (mucinous type and intestinal or colonic type). In the absence of explicit surgical guidelines for the management of appendix cancer, management is largely guided by retrospective series [1,9]. The question of whether a simple appendectomy is enough or if a hemicolectomy is needed remains controversial. A study that utilized the SEER database proposed a nomogram to predict five-year survival and guide the management of adenocarcinoma of the appendix; it suggested that depth of invasion should be used as a guide to the surgical intervention. The study showed that for tumors limited to the mucosa, there was no difference in the overall five-year survival between patients who underwent local tumor excisions, such as appendectomy, and those who chose extended surgery (hemicolectomy and more; p=0.752). On the other hand, for tumors that invaded the mucosa, patients who underwent extended surgery were found to have better overall survival compared to those who only underwent localized resection (p=0.011 for tumor invading the serosa, and p=0.956 for tumor that had invaded the serosa) [1]. Other studies suggested a right hemicolectomy as the recommended surgical intervention for all SRCCA [10]. Our analysis showed a five-year CSS of 26.5% for subtotal colectomy compared to 20.9% for partial colectomy (including appendectomy) with a p-value of <0.001.

The role of adjuvant chemotherapy and radiotherapy in appendiceal adenocarcinoma in general, and in SRCCA in particular, remains uncertain. A retrospective study conducted at The University of Texas MD Anderson Cancer Center that involved 142 patients with poorly differentiated and signet ring cell adenocarcinomas of the appendix showed that systemic chemotherapy appears to be a viable treatment option for patients with metastatic disease (HR: 0.5; p=0.02) [11].

Cytoreductive surgery and hyperthermic intraperitoneal chemotherapy (CRS/HIPEC) is the standard treatment for peritoneal dissemination (PD) from appendiceal cancer. PD of the SRCC is termed peritoneal mucinous carcinomatosis with signet ring cells (PMCA-S). In a retrospective study carried out at the Wake Forest Baptist Health to determine the significance of signet ring cells in mucinous adenocarcinoma of the peritoneum from the appendiceal origin, signet ring cells were identified in 29 of 55 cases. All of the 29 cases were high grade. A significant survival difference was seen for cases of high-grade mucinous adenocarcinoma with signet ring cells invading tissue with a median overall survival of 0.5 years versus 2.9 years for cases of high-grade mucinous adenocarcinoma without signet ring cells (p=0.04), and 2.4 years for high-grade mucinous adenocarcinoma with signet ring cells within mucin pools (p=0.03) [12].

Given the poor prognosis of PD from SRCCA, the benefit of CRS/HIPEC in such cases has been questioned and results of the previous retrospective studies in that regard were not consistent. A retrospective study included 196 patients with PD from high-grade appendiceal malignancies, of whom 151 underwent successful CRS/HIPEC procedures, of which 82 % had a signet cell component. The study showed that CRS/HIPEC can achieve a five-year survival of 25% for a patient with PMCA-S, which may improve to 51% with negative lymph nodes. The study endorsed the treatment method [13]. On the other hand, in another retrospective study conducted at the Washington Cancer Institute that included 494 patients with PMCA, of whom 80 had PMCA-S, the five-year overall survival for the latter group was 22% and the median survival time was 18.9 months. The independent predictors for a poor overall survival included incompleteness of cytoreduction, PMCA-S histomorphology, and distant metastasis [14].

The management of SRCCA is mired in controversies, and many questions remain unanswered. Further research with a larger cohort of patients is needed to address these knowledge gaps regarding the management of SRCCA and to provide the most optimal treatment for the patients.

A major limitation of our study was its retrospective design. Data compiled in the database may have been incompletely, improperly, or inaccurately recorded from the patients’ charts. Additionally, selection bias was inherent as only patients from certain registries were added. Finally, although stage reporting was done using two TNM AJCC staging editions (third and sixth) according to the year of diagnosis, authors do not believe that it has affected the results of the analysis as the two staging editions have the same TNM components for colonic cancers (appendicular cancers were not a separate diagnosis in these two editions), except for N3, which was considered in the third edition but no longer used in the sixth.

## Conclusions

In this study, we discussed the various features and survival rates of patients diagnosed with SRCCA. SRCCA is a rare tumor with a high prevalence among old-aged white females. This tumor is usually diagnosed in an advanced stage and has a dismal prognosis. Surgical intervention, regardless of the extent, showed better survival probabilities compared to no surgery.
